# Insomnia

**Published:** 1890-07

**Authors:** 


### INSOMNIA.

Extract from an article in the Medical Press and Circular, by EDWARD WARREN BEY, M. D., C. M., LL. D., D. M. P., Chevalier of the Legion of Honour, 15 Rue Caumartin, Paris:

To those familiar with the use of BROMIDIA (Battle), no argument is neccssary, for it speaks for itself, by fulfilling the indications for which it is administered with a certainty, efficiency and harmlessness, which elicit at once the wonder of the patient and the delight of the prescriber, and give to the profession the assurance of possessing one remedy at least which approximates so near to infallibility of action as to justify the title of specific.

